# Antimicrobial resistance burden pre and post-COVID-19 pandemic with mapping the multidrug resistance in Egypt: a comparative cross-sectional study

**DOI:** 10.1038/s41598-024-56254-4

**Published:** 2024-03-26

**Authors:** Shaimaa Abdelaziz Abdelmoneim, Ramy Mohamed Ghazy, Eman Anwar Sultan, Mahmoud A. Hassaan, Mohamed Anwar Mahgoub

**Affiliations:** 1 Medical Research Institute, Alexandria University, Alexandria, Egypt; 2 Clinical Research Administration, Alexandria Directorate of Health Affairs, Egyptian Ministry of Health and Population, Alexandria, Egypt; 3 Family and Community Medicine Department, College of Medicine, King Khalid University, Abha, Saudi Arabia; 4 Tropical Health Department, High Institute of Public Health, Alexandria University, Alexandria, Egypt; 5 Community Medicine Department, Faculty of Medicine, Alexandria University, Alexandria, Egypt; 6 Institute of Graduate Studies and Research, Alexandria University, Alexandria, Egypt; 7 Department of Microbiology, High Institute of Public Health, Alexandria University, Alexandria, Egypt

**Keywords:** Antimicrobial resistance, MDR, XDR, COVID-19, Egypt, Spatial distribution, Antimicrobials, Microbial communities, Health care, Medical research

## Abstract

Overuse of antibiotics during coronavirus disease 2019 (COVID-19) in an attempt to reduce COVID-19 mortality in the short term may have contributed to long-term mortality from antimicrobial resistance (AMR). The aim of this study was to evaluate the impact of the COVID-19 pandemic on AMR in Egypt and map the distribution of multidrug-resistant (MDR) and extensive drug-resistant (XDR) across Egypt. Through a multicenter cross-sectional study 2430 culture results were collected in 2019 and 2022 pre and post-COVID-19 pandemic in Egypt, including 400 *Klebsiella pneumoniae,* 760 *Escherichia coli*, 650 *Acinetobacter baumannii*, and 620 *Methicillin-resistant* *staphylococcus aureus* (MRSA) culture results. MDR and XDR culture results distribution across Egypt was highlighted through the geographic information system. Mixed effect logistic regression models and sub-group analysis were performed according to the type of specimens to test the impact of COVID-19 on resistance. Adjusted analysis demonstrated *K. pneumoniae* resistance has increased against quinolones and carbapenems (*P* < 0.001). Resistance of *E. coli* has increased significantly against imipenem and meropenem. While *E.coli* susceptibility has increased to cefoxitin, levofloxacin, and ciprofloxacin. *A. baumannii* resistance has increased more than double against ceftazidime, cefepime, and piperacillin-tazobactam (*P* < 0.001). MRSA reserved its susceptibility to vancomycin and linezolid. MDR *K. pneumoniae* and *A. baumannii* have increased post-COVID-19 from 67% to 94% and from 79% to 98%, respectively (*P* < 0.001). XDR *K. pneumoniae* and *A. baumannii* have increased from 6% to 46%, and from 47% to 69%, respectively (*P* < 0.001). COVID-19 has changed the profile of AMR in Egypt so that urgent action is required to mitigate this threat and preserve our capacity to face infections in future decades.

## Introduction

Antimicrobial resistance (AMR) is a natural phenomenon, however, antibiotic misuse is accelerating it. The AMR burden resulted in 1.27 million deaths in 2019 and was predicted to result in more than 10 million deaths in 2050, as well as its economic burden^1^. Although it is an emerging problem, the world's attention was diverted away from AMR during the coronavirus disease 2019 (COVID-19) pandemic^2^. On March 11, 2020, the World Health Organization (WHO) declared COVID-19 pandemic. However, if we don't change our behavior towards using antibiotics, AMR could also become a pandemic in a gradual process^2^. During COVID-19, due to the high admission rates and broad-spectrum antibiotic use, there was increased risk of hospital-acquired infections (HAIs) with multidrug-resistant (MDR) organisms^3,4^.

Overuse of antibiotics during COVID-19 in an attempt to reduce COVID-19 mortality in the short term may have contributed to long-term mortality from AMR^5^. Although the Infectious Diseases Society of America (IDSA) recommended against the use of antibiotics without secondary infection and states that only 8% of patients with COVID-19 developed secondary bacterial superinfections requiring antibiotics, the administration rate of broad-spectrum antibiotics reached 72% in patients with COVID-19^6^. Several studies have shown that AMR has increased for different types of bacteria e.g., *Escherichia coli* (*E. coli*), *Klebsiella pneumoniae* (*K. pneumoniae*), *staphylococcus aureus*, and *Acinetobacter baumannii* (*A. baumannii*) after the COVID-19 pandemic^7–9^. Recent meta-analyses have revealed that during COVID-19, the incidence of multidrug resistance (MDR) has increased significantly, with rates ranging from 24% to 37.5%^10,11^.

During the COVID-19 pandemic, the misuse of antibiotics in Egyptian hospitals may have occurred due to the lack of biological markers to distinguish between viral and bacterial infections. This made it difficult to identify the appropriate treatment for patients^6^. Moreover, 45.9% of the Egyptian population practiced self-medication and used antibiotics without a prescription^7^. Several studies investigating AMR before the COVID-19 pandemic in 2000 and 2011 reported high rates of *Methicillin-resistant Staphylococcus aureus* (MRSA) and ceftazidime resistance among *E.coli*, *K. pneumoniae* and *Enterobacter* species^12,13^, but to our knowledge, there is no study assessing the impact of COVID-19 on AMR post COVID-19 pandemic.

This study hypothesized an increased rate of AMR during the COVID-19 pandemic in Egypt. The aim of this study was to investigate the impact of the COVID-19 pandemic on the spread of AMR in Egypt. In addition to determine the frequency of bacteria that are resistant to multiple drugs, including extensively drug-resistant bacteria (XDR) and pan drug-resistant bacteria (PDR). Furthermore, the study aimed to analyze the temporal and geospatial patterns of these bacteria.

## Methods

According to the latest census in 2024, Egypt's population has increased by 1.57% to reach 114,484,252. They reside in 27 governorates across the country^14^. Through a multicenter cross-sectional study, culture results were collected from the Mabaret Al-Asafra laboratories database, one of the largest laboratories across Egypt, and other laboratories dealing with Mabaret Al-Asafra laboratories (lab to lab) in sixteen different governorates across Egypt. Each laboratory receives more than 30,000 cultures per year. On February 14, 2020 Egypt reported the first COVID-19 case. Positive culture results were collected over six months before and after the COVID-19 pandemic, throughout 2019 and 2022. We excluded the polymicrobial cultures and positive fungal cultures results. Culture results were collected over different seasons and different types of specimens; respiratory, blood, pus, and urine cultures. Antimicrobial susceptibility testing followed The Clinical and Laboratory Standards Institute (CLSI) guidelines^15^. The most prevalent bacteria in Egypt; *K. pneumoniae, E.coli, A. baumannii*, and MRSA were selected to evaluate AMR^16,17^. A total of 2430 culture results were collected over the two periods (pre and post COVID-19) including 400 culture results of *K*. *pneumoniae*, 650 culture results of *A*. *baumannii,* 760 culture results of *E.coli*, and 620 culture results of MRSA, divided according to 1:1 ratio before and after COVID-19 pandemic.

### Outcomes

The main objective of this study was to compare AMR pre-and post-COVID-19 and exploring the MDR, XDR, and PDR distribution post COVID-19 in Egypt. We also described the temporal and geospatial patterns of AMR.

### Operational definition^18^

- Multidrug resistant bacteria (MDR) are bacteria resistant to at least one agent in three or more antimicrobial categories.
- Extensively drug-resistant (XDR): this means that bacteria are only susceptible to one or two antimicrobial categories.
- Pan drug-resistant (PDR): are bacteria that are resistant to all agents in all antimicrobial categories.

### Ethical approval

The ethical approval was received from the Ethical Committee of the Faculty of Medicine, Alexandria University (IRB number: 00012098, serial number: 0305804). Informed consent was waived, as the culture results were collected anonymously, without identifying any patient characteristics, from the laboratory electronic database. All methods were performed in accordance with the relevant guidelines and regulations of the ethical committee of the Faculty of Medicine, Alexandria University.

### Data management

Culture results were described as the type of specimen (blood, sputum, urine, and pus), seasons, geographical distribution (Metropolitan, Upper Egypt, and Lower Egypt), type of AMR (MDR, XDR, and PDR) in percentages, and the prevalence of resistance and sensitivity of each organism in percentages.

### Statistical analysis plan

AMR was compared pre- and post-COVID-19 pandemic using chi-square test for each type of tested antibiotic. Subgroup analysis was performed for each bacterial species according to the type of specimens, with at least 10 cultures in each period. Mixed-effects logistic regression models were used to test the impact of COVID-19 on resistance while controlling the type of specimens, seasons, and geographical distribution. Intermediate sensitivity was excluded from the logistic regression. Geographical distribution was used as a random intercept, and the period pre- or post-COVID-19, type of specimens, and seasons were the fixed effects. Models were tested for logistic regression assumptions and goodness of fit; the best models were selected according to the lowest Akaike information criterion (AIC). Results of logistic regression were represented as odds ratio (OR, 95%CI). Antibiotics were selected for logistic regression according to the IDSA 2023 Guidance and the most commonly used antibiotics in Egypt^19^. Geographic information system (GIS) was used in mapping MDR and XDR, thus providing an insight into the distribution pattern and representing the spatial variations in MDR and XDR pre- and post-COVID-19 pandemic. For this purpose, a geodatabase was developed to integrate relevant data on MDR and XDR before and after COVID-19. Generally, GIS application in mapping MDR and XDR organisms can inform decision-making, resource allocation, and public health interventions. This, consequently, can contribute to controlling the spread of AMR.

## Results

### Pattern of AMR before and after COVID-19

Results illustrated significant differences in the number of positive cultures pre and post the COVID-19 pandemic for each type of specimen, among the cultures of *K. pneumoniae* (*P* < 0.001), *A. baumannii* (*P* < 0.001), *E.coli* (*P* < 0.001), and MRSA (*P* = 0.002). *K. pneumoniae* showed the highest prevalence in urine specimens (56.0% pre-COVID-19 pandemic and 71.0% in blood cultures post-COVID-19 pandemic). *A. baumannii* showed the highest prevalence in respiratory culture results (59.0% before the COVID-19 pandemic and 65.0% in blood samples after the COVID-19 pandemic). The prevalence of *E. coli* in urine cultures was high before and after the COVID-19 pandemic (86.0% and 74.0%, respectively). MRSA showed the highest prevalence among pus samples before and after the COVID-19 pandemic (89.0% and 80.0%, respectively) Supplementary Table S1.

#### Temporal and geographical pattern of AMR

The temporal cultures' distribution among the different seasons pre- and post-COVID-19 pandemic didn’t show a significant difference in *K. pneumoniae* (*P* = 0.612) or *A. baumannii* (*P* = 0.203), although the distribution of *E.coli* cultures and MRSA were significantly different (*P* < 0.001). The geographical distribution of the cultures in Egypt was significantly different post-COVID-19 pandemic for *A. baumannii* (*P* = 0.024), E.coli (*P* = 0.012), and MRSA (*P* < 0.001), and non-significant for *K. pneumoniae* (*P* = 0.751) Supplementary Table S1.

### Difference between pre and post COVID-19 pandemic

**Figure 1:**
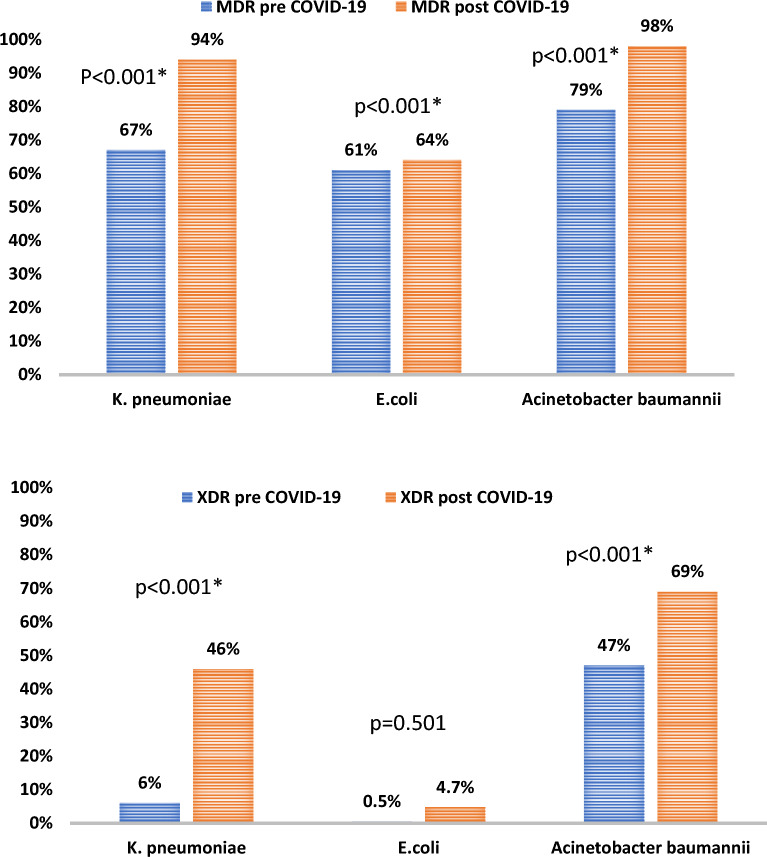
MDR and XDR pre and post COVID-19.

Extended spectrum beta-lactamase (ESBL) producing *K. pneumoniae* represented (62.1%) of *K. pneumoniae* cultures before COVID-19 and 37.9% after COVID-19 (*P* = 0.004). ESBL producing *E. coli* represented 43.7% of *E.coli* culture results pre-COVID-19 and 49.7% post COVID-19 (*P* = 0.240). Both MDR and XDR *A. baumannii* before and after COVID-19 increased significantly (*P* < 0.001). Similarly, MDR and XDR *K. pneumoniae* showed a significant increase after COVID-19 (*P* < 0.001). Of note, XDR *E.coli* did not show a significant increase after COVID-19, (*P* = 0.501), although MDR *E.coli* showed a significant increase (*P* < 0.001). Figure 1

### K. pneumoniae

Cultures of *K. pneumoniae* showed an increase in resistance against most antibiotics tested after the COVID-19 pandemic, amikacin 65.0%, amoxicillin-clavulanate 90.0%, ampicillin-sulbactam (SAM) 92.0%, cefepime 87.0%, cefotaxime 88.0%, cefoxitin 75.0%, ceftazidime 88.0%, ceftriaxone 88.0%, ciprofloxacin 70.0%, doxycycline 95.0%, ertapenem 69.0%, gentamycin 70.0%, imipenem 65.0%, levofloxacin 69.0%, meropenem 66.0%, moxifloxacin 70.0%, ofloxacin 71.0%, piperacillin/tazobactam (TZP) 71.0%, and trimethoprim/sulfamethoxazole (SXT) 71.0% (*P* < 0.001). Resistance against nitrofurantoin (21.0%) and fosfomycin (18.0%) decreased significantly. Tigecycline, colistin, and ceftazidime-avibactam were not tested for most of the cases and were excluded from the analysis Supplementary Table S2.

For *K. pneumoniae*, a mixed effects logistic regression model revealed an increase in resistance post-COVID-19 pandemic for doxycycline 1.15 (95% CI 1.07–1.21), ceftazidime 1.16 (95% CI 1.08–1.24), cefepime 1.16 (95% CI 1.08–1.24), amikacin 1.27 (95% CI 1.19–1.34), levofloxacin 1.18 (95% CI 1.09–1.28), ciprofloxacin 1.16 (95% CI 1.07–1.25), moxifloxacin 1.19 (95% CI 1.10–1.28), ertapenem 1.23 (95% CI 1.15–1.31), imipenem 1.18 (95% CI 1.11–1.26), and meropenem 1.20 (95% CI 1.12–1.27). Nitrofurantoin was tested only for urine samples, where *K. pneumoniae* showed a significant increase in resistance against nitrofurantoin with an OR of 1.29 (95% CI 1.15–1.44) Supplementary Table S3.

**Figure 2:**
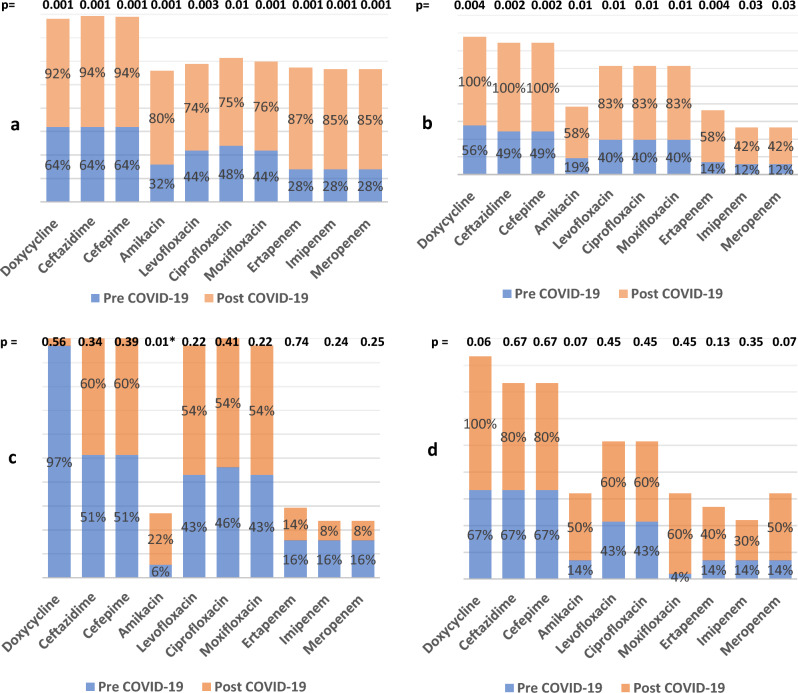
Sub-group analysis for *K. pneumoniae* resistance according to type of specimen, (**a**) blood, (**b**) respiratory specimen, (**c**) urine, (**d**) pus.

Sub-group analysis revealed that *K. pneumoniae* resistance in blood and sputum specimens significantly increased against most of the tested antibiotics. In the urine sample, *K. pneumoniae* resistance increased significantly against amikacin and non-significantly against quinolones, doxycycline, cefepime, and ceftazidime. While resistance decreased non-significantly against carbapenems. Pus specimens did not show any significant change in resistance after COVID-19. Figure 2

**Figure 3:**
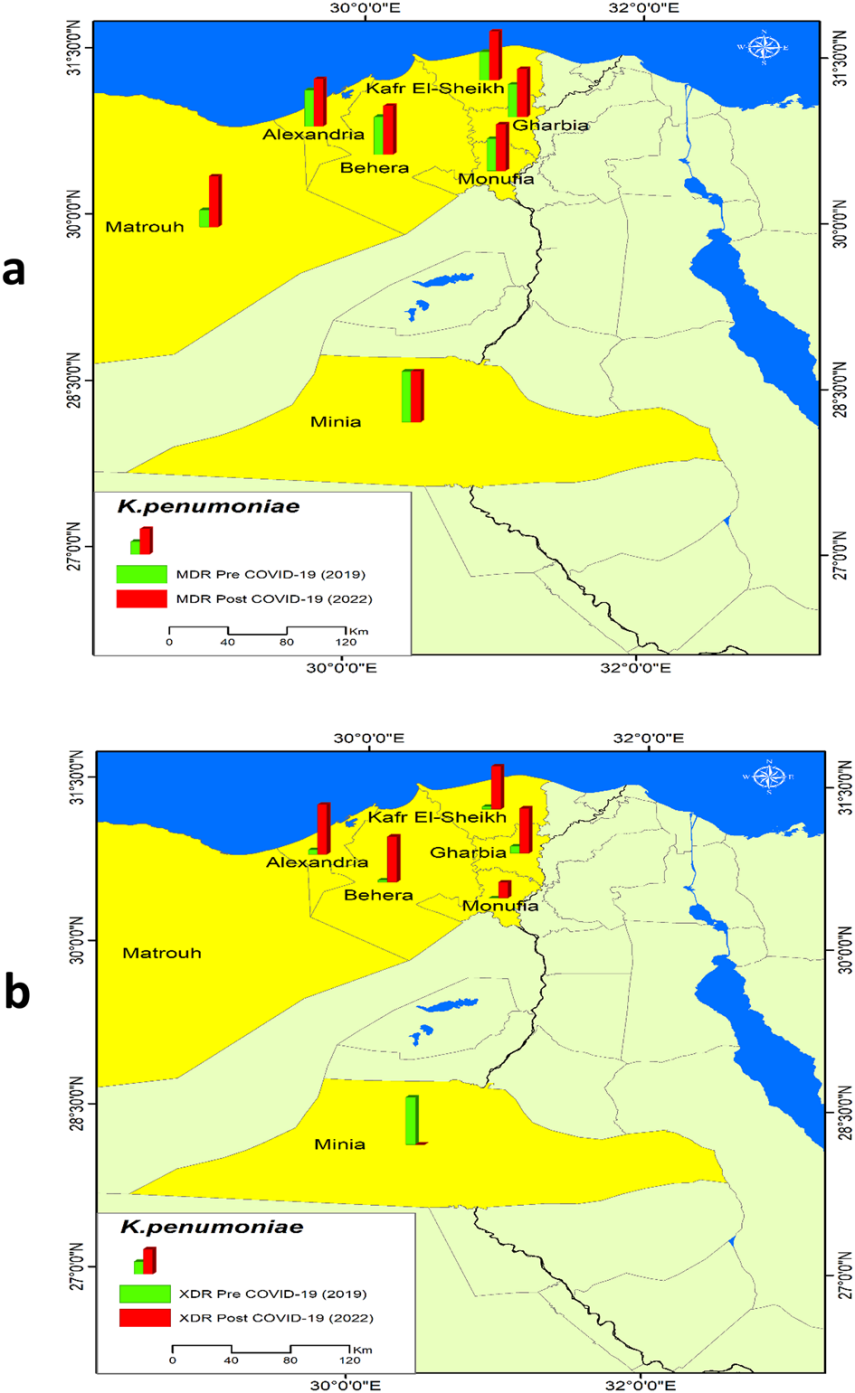
Distribution of *K. pneumoniae* in the sample pre and post COVID-19, (**a**) *K. pneumoniae* MDR , (**b**) *K. pneumoniae* XDR.

Figure 3 shows the MDR and XDR *K. pneumoniae* spatial distribution among the sample. It illustrates that MDR increased in six governorates post-COVID-19, Alexandria, Matrouh, Behera, Kafr Elsheikh, Monufia, and Gharbia; but there was no change in the MDR pattern in one governorate (Minia). XDR increased in five governorates, Alexandria, Behera, Kafr Elsheikh, Monufia, and Gharbia; but decreased in one governorate (Minia).

### A. baumannii

*A. baumannii* resistance had increased after COVID-19 pandemic for amikacin 94.2%, SAM 93.5%, cefepime 98.0%, ceftazidime 99.1%, ceftriaxone 99.1%, ciprofloxacin 88.9%, doxycycline 92.6%, gentamicin 83.7%, imipenem 84%, levofloxacin 87.7%, meropenem 89.2%, TZP 95.0%, SXT 95.0% (*P* < 0.001), and cefotaxime 99.1% (*P* = 0.003). *A. baumannii* did not show a significant increase in resistance for colistin 0.3% and tigecycline 2.2% (*P* = 0.316), respectively. Supplementary Table S4.

The mixed-effects logistic regression model for *A. baumannii* resistance showed a significant increase in resistance post-COVID-19 against doxycycline 1.73 (95% CI 1.72–1.73), ceftazidime 2.75 (95% CI 1.86–4.07), cefepime 2.26 (95% CI 1.70–3.01), levofloxacin 1.36 (95% CI 1.16–1.59), ciprofloxacin 1.42 (95% CI 1.20–1.68), TZP 2.13 (95% CI 2.12–2.13), amikacin 1.82 (95% CI 1.81–1.84), imipenem 1.48 (95% CI 1.48–1.49), and meropenem 1.51 (95% CI 1.50–1.51).Supplementary Table S5.

**Figure 4:**
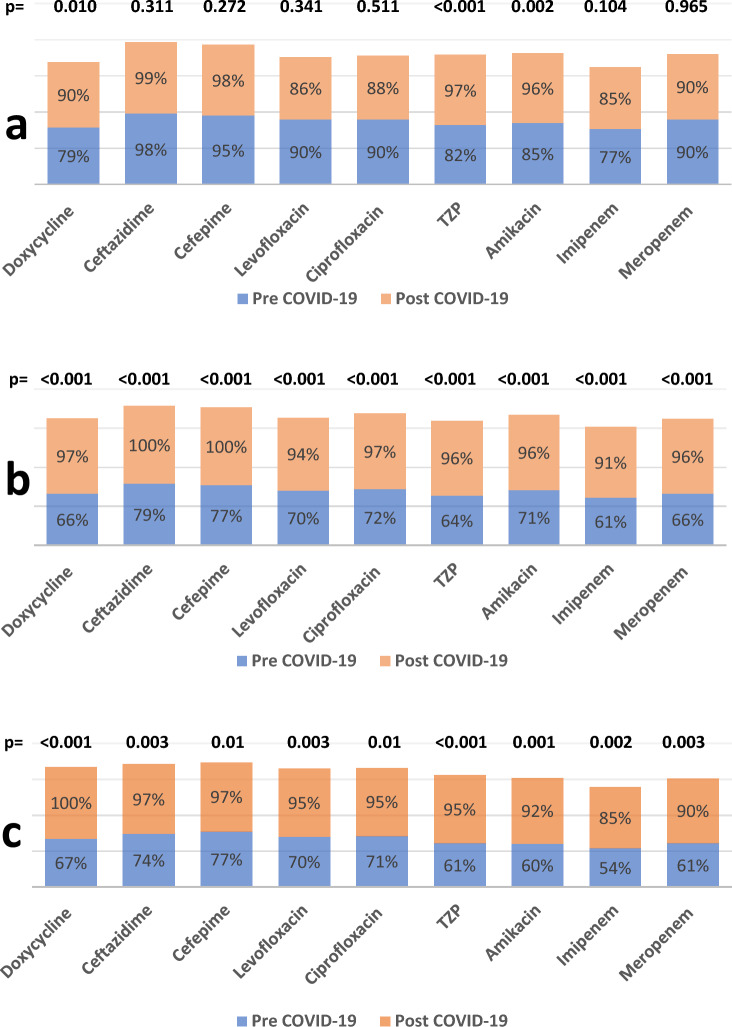
Sub-group analysis for *A. baumannii* resistance according to the type of specimen. (**a**) blood, (**b**) sputum, (**c**) pus

The subgroup of *A. baumannii* samples revealed that the sputum and pus culture results showed increased resistance to most antibiotics tested. On the other hand, blood culture results revealed a non-significant increase in resistance except against amikacin from 85.0% to 96.0% (*P* = 0.002), TZP from 82.0% to 97.0% (*P* < 0.001), and doxycycline from 79.0% to 90.0% (*P* = 0.010),Fig. 4

**Figure 5:**
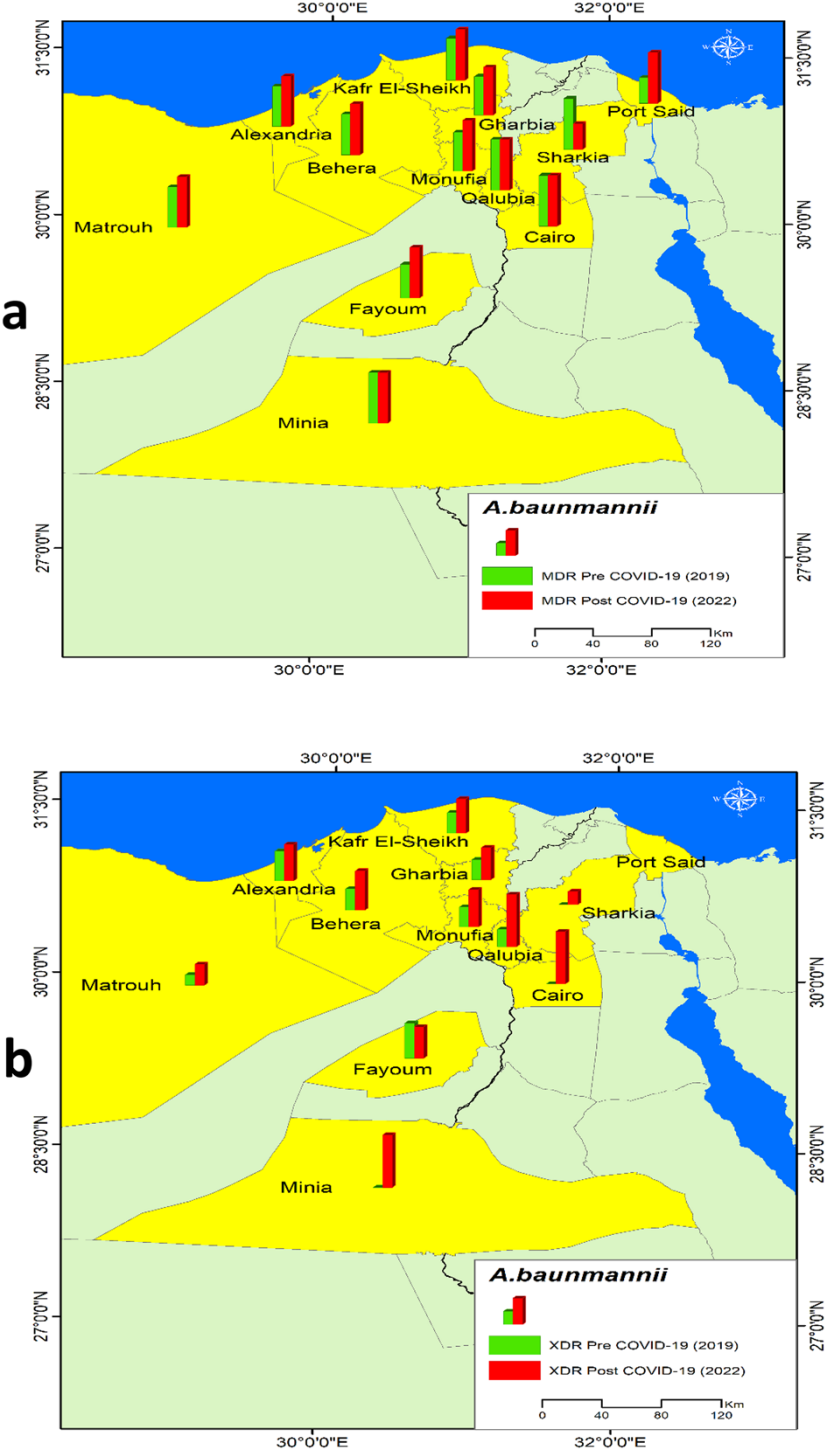
Distribution of *A. baumannii* in the sample pre and post COVID-19, (**a**) *A. baumannii* MDR ,(**b**) *K. pneumoniae* XDR.

Figure 5 shows the distribution of MDR and XDR *A. baumannii* among the sample. The MDR increased post-COVID-19 in eight governorates but didn’t change in Cairo, Qalubia, and Minia, while it decreased in Al-sharkia. The XDR increased post-COVID-19 in ten governorates, it only decreased in Fayum.

### E.coli

There was an increase in *E. coli* resistance after COVID-19 against amikacin 8.0%, amoxicillin-clavulanate 66.0%, SAM 66.0%, cefotaxime 58.0%, ceftriaxone 59.0%, ceftazidime 59.0%, cefepime 58.0%, doxycycline 98.0%, ertapenem 7.0%, fosfomycin 50.0%, gentamicin 24.0%, imipenem 6.0%, meropenem 6.3%, nitrofurantoin 46.0%, piperacillin/tazobactam 24.0%, and SXT 67.0%. *E. coli* resistance significantly decreased against ciprofloxacin (37.4%), levofloxacin (37.0%), norfloxacin (23.0%), and ofloxacin (38.4%). Otherwise, resistance against cefoxitin and moxifloxacin showed no significant change post-COVID-19. Supplementary Table S6.

The mixed effects logistic regression model for *E. coli* resistance revealed a significant increase in resistance against fosfomycin 1.44 (95% CI 1.37–1.50), nitrofurantoin 1.28 (95% CI 1.21–1.34), SAM 1.11 (95% CI 1.06–1.11), ceftriaxone 1.08 (95% CI 1.03–1.14), ceftazidime 1.09 (95% CI 1.03–1.14), cefepime 1.08 (95% CI 1.03–1.14), imipenem 1.03 (95% CI 1.01–1.05), and meropenem 1.03 (95% CI 1.01–1.05). There was a non-significant increase in resistance against TZP 1.02 (95% CI 0.97–1.07) and cefoxitin 0.97 (95% CI 0.93–1.02). Although *E. coli* resistance decreased significantly against levofloxacin 0.94 (95% CI 0.89–0.98) and ciprofloxacin 0.93 (95% CI 0.89–0.98). Supplementary Table S7.

**Figure 6:**
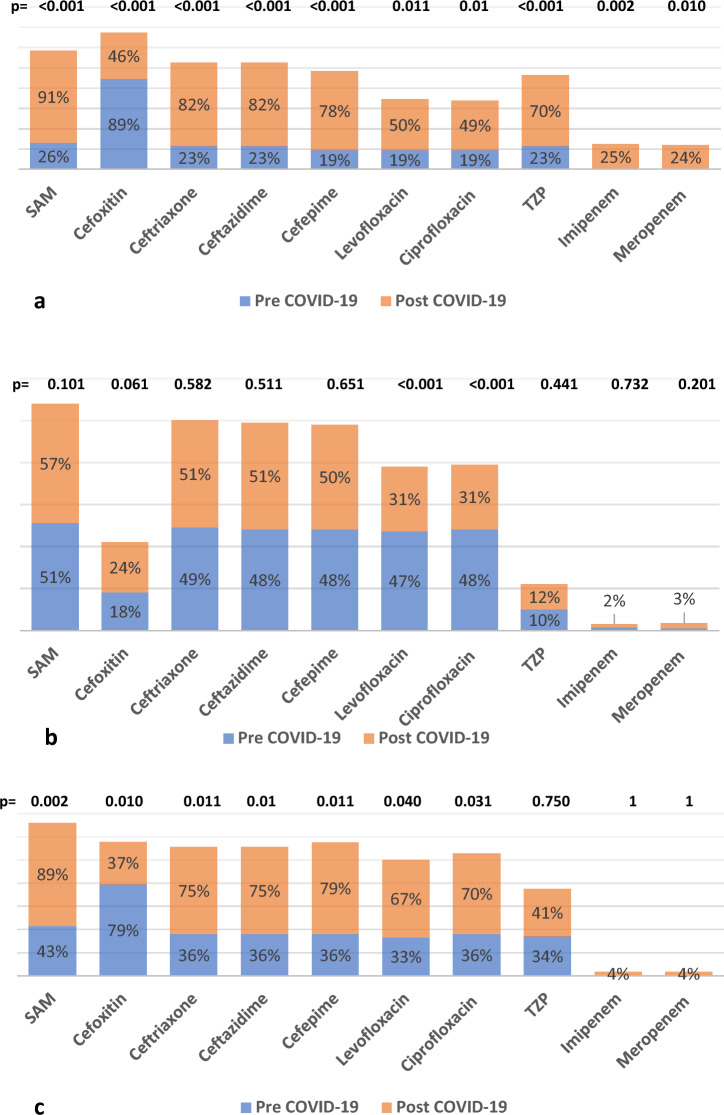
Sub-group analysis for E.coli resistance according to the type of specimen. (**a**) blood, (**b**) urine, (**c**) pus

The subgroup analysis of the *E. coli* blood culture results showed a significant increase in resistance against most tested antibiotics except cefoxitin, where the *E. coli* blood culture results have become more susceptible to cefoxitin post COVID-19 pandemic, where resistance against cefoxitin decreased from 89.0% to 46.0% (*P* < 0.001). The results of the urine culture of *E. coli* showed a non-significant increase in resistance against most tested antibiotics; however, the *E. coli* in the urine has become more susceptible to levofloxacin and ciprofloxacin, where resistance decreased from 47.0% to 31.0% and from 48.0% to 31.0% (*P* < 0.001), respectively. The results of the *E. coli* pus culture showed a significant increase in resistance against most tested antibiotics and a non-significant increase against TZP, imipenem, and meropenem. *E. coli* in pus species have become more susceptible to cefoxitin, where resistance decreased from 79.0% to 37.0% (*P* = 0.010).Fig. 6

**Figure 7:**
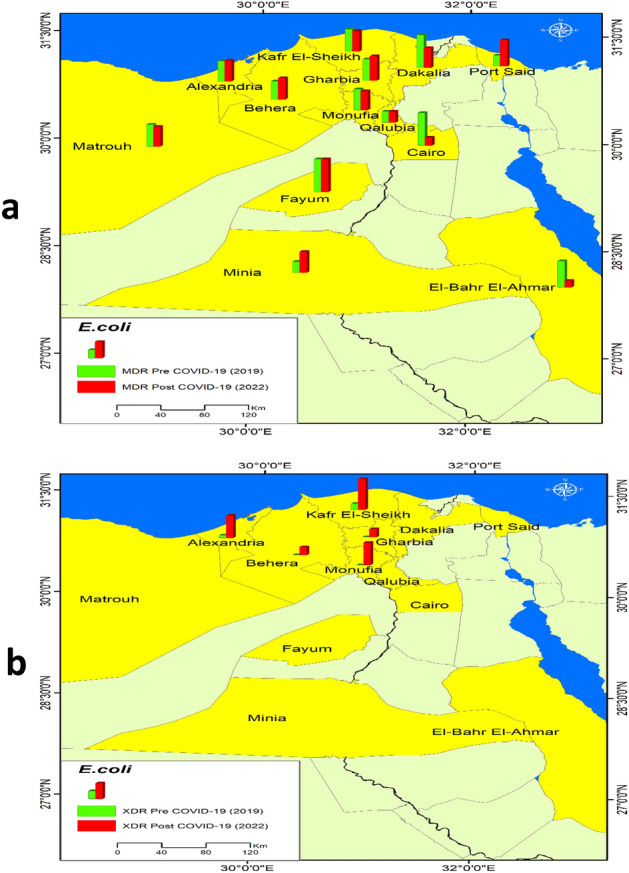
Distribution of E.coli in the sample pre and post COVID-19, (**a**) E.coli MDR, (**b**) E.coli XDR.

Figure 7 shows the distribution of MDR and XDR *E. coli* among the sample. It illustrates an increase in MDR *E. coli* after COVID-19 pandemic in Alexandria, Behera, Gharbia, Port Said, and Minia while decreasing in MDR in Cairo, El Bahr Elahmar, Dakahlia, Monufia, Kafr El-Sheikh, and Matrouh. But there was no change in MDR in Fayum and Qalubia. XDR *E. coli* increased in Alexandria, Behera, Kafr El-Sheikh, Monufia, and Gharbia.

### MRSA

There were significant differences in resistance after COVID-19 to azithromycin (43.2%), clindamycin (38.7%), doxycycline (69.4%), levofloxacin (20.0%), and moxifloxacin (20.0%). On the contrary, MRSA did not show a significant change in resistance after COVID-19 against linezolid, tigecycline, ciprofloxacin, gentamicin, ofloxacin, SXT, and vancomycin. Supplementary Table S7.

Mixed effects for the logistic regression model of MRSA resistance revealed a significant increase in resistance against clindamycin 1.07 (95% CI 1.05–1.110), doxycycline 1.15 (95% CI 1.12–1.18), levofloxacin 1.02 (95% CI 1.00–1.04), and moxifloxacin 1.03 (95% CI 1.01–1.05). Supplementary Table S8.

**Figure 8:**
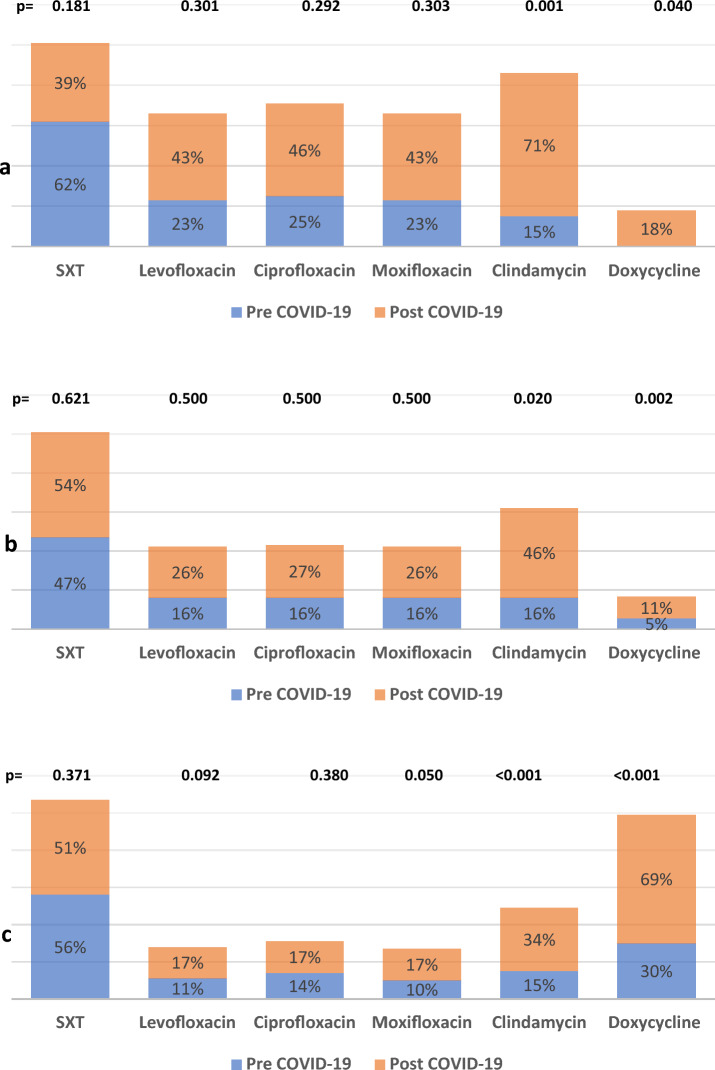
Sub-group analysis for MRSA resistance according to the type of specimen. (**a**) blood, (**b**) sputum, (**c**) pus

The subgroup analysis for MRSA revealed that resistance in blood samples increased significantly against clindamycin from 15.0% to 71.0% (*P* = 0.001) and developed resistance against doxycycline from 0% to 18.0% (*P* = 0.040). Resistance against SXT decreased from 62.0% to 39.0%, but this decrease was not statistically significant (*P* = 0.181). There was a slight increase in resistance against levofloxacin, ciprofloxacin, and moxifloxacin, although it was not statistically significant (*P* = 0.301), (*P* = 0.292), and (*P* = 0.303), respectively. The MRSA in the sputum showed an increase in resistance against clindamycin from 15.0% to 46.0% (*P* = 0.020) and doxycycline from 5.0% to 11.0% (*P* = 0.002). There was a non-significant increase in resistance against quinolones (*P* = 0.500) and SXT (*P* = 0.621). There was a significant increase in the resistance of MRSA in pus specimens against clindamycin. Resistance increased from 15.0% to 34.0% (*P* < 0.001). Similarly, doxycycline resistance increased from 30.0% to 69.0% (*P* < 0.001). However, MRSA resistance in pus did not increase significantly against quinolones and SXT Figure 8.

## Discussion

The aim of this study was to detect the impact of COVID-19 on AMR in Egypt and to detect the distribution of MDR and XDR in Egypt as an opportunity to face the threat of AMR. The current study reported a significant difference in the number of positive specimens pre- and post-COVID-19 pandemic for *K. pneumoniae, A. baumannii, E. coli*, and MRSA cultures. The resistance of *K. pneumoniae, A. baumannii, E. coli*, and MRSA against various antibiotics increased after COVID-19. The distribution of cultures between different seasons before and after the COVID-19 showed significant differences for positive *E. coli* cultures and MRSA, but not for *K. pneumoniae* or *A. baumannii*. Geographical distribution of cultures in Egypt significantly differed post-COVID-19 pandemic for *A. baumannii, E. coli*, and MRSA, but not for *K. pneumoniae*.

MDR and XDR distribution maps illustrated changes in resistance patterns across various governorates post-COVID-19. Similar to our findings, multiple studies highlighted the impact of the COVID-19 pandemic on the increase, in AMR. Sulayyim^20^ illustrated that AMR has increased during COVID-19, especially for *A. baumannii*, *K. pneumoniae*, and *Staphylococcus aureus*. Serapide^21^ also observed a higher AMR among COVID-19 patients, with 67.8% Gram-negative culture results were classified as MDR.

Respecting to impact of climate change on AMR, where heat and humidity are considered risk factors for AMR^22^, In our study, seasonality was considered as a variable in logistic regression, however, COVID-19 had the greatest effect on AMR in our study regardless the seasonality.

### K. pneumoniae

Our study revealed that resistance of K. pneumoniae has increased against 20 antibiotics post COVID-19 pandemic. Previous studies concurred with our results, Nicola et al.^23^ described an outbreak of a ceftazidime-avibactam-resistant strain of *K. pneumoniae* during COVID-19, indicating the emergence of increased resistance against a new approved combination during COVID-19. A study in Egypt reported that *K. pneumoniae* became 100% resistant to tigecycline and more than 65% resistant to cephalosporins and fluoroquinolones^24^.

### A. baumannii

Our results illustrated that the resistance increased significantly against 14 antibiotics, amikacin, ampicillin/sulbactam, cefepime, cefotaxime, ceftazidime, ceftriaxone, ciprofloxacin, doxycycline, gentamycin, imipenem, levofloxacin, meropenem, TZP, and SXT. Resistance to tigecycline and colistin did not increase significantly. Previous studies agreed with our findings, where Kinross et al.^25^ concluded that resistance to *A. baumannii* has increased during COVID-19. A 57% increase in carbapenem-resistant *A. baumannii* bloodstream infections was reported in European hospitals from 2020 to 2021 compared to 2018–2019. Moreover, Sayed et al.^26^, reported that the risk of carbapenem resistant *A. baumannii* infections increased to 92.7% among COVID-19 patients.

### E.coli

Multiple studies show that antibiotic resistance in *E. coli* culture results has increased over time, especially during the COVID-19 pandemic^24,27^. Wardoyo^27^ *E. coli*, showed resistance against penicillin, ampicillin, and cefixime at different rates of 100%, 97%, and 89%, respectively^24^. Our findings aligned with previous findings, although susceptibility to quinolones and cefoxitin increased. While *E. coli* resistance in urine culture results increased against fosfomycin by 44% and nitrofurantoin by 28%.

### MRSA

Our results illustrated a significant increase in resistance to MRSA against five antibiotics, azithromycin, clindamycin, doxycycline, levofloxacin, and moxifloxacin with reserved susceptibility to vancomycin and linezolid. The adjusted analysis showed a significant increase in resistance against clindamycin, doxycycline, levofloxacin moxifloxacin, but not against ciprofloxacin. In the same vein, Helmy et al.^24^ reported that the MRSA culture results had low resistance rates against tigecycline and linezolid. Furthermore, a meta analysis included 64 studies concluded that resistance of MRSA slightly changed post COVID-19 pandemic, with reserved susceptibility to vancomycin, tigecycline, or linezolid^28^.

### MDR and XDR

During COVID-19 pandemic, MDR bacteria were implicated in 73.17% of COVID-19 patients with secondary infection. *K. pneumoniae, Enterococcus* spp*.* and MRSA were the most common MDR detected in 20.43%, 4.30% and 4.30% of all culture results, respectively^22^. In the current study, MDR and XDR but not PDR have increased significantly in Egypt post COVID-19 pandemic. In the same vein, Pałka et al.^29^ demonstrated high prevalence of XDR organisms of 22.6% in the intensive care unit (ICU) and 14.8% in non-ICUs among all isolate especially XDR-*A. baumannii*. Increasing MDR post COIVD-19 might be linked to human factors including delayed care, increasing device usage, and inappropriate use of antibiotics in the trial of treat the complications of COVID-19 pandemic^3,30^. The disparity in the distribution of MDR and XDR in Egypt before and after the pandemic could be attributed to the different AMR response plans in each governorate, highlighting the importance of developing a national AMR response plan for Egypt. The findings of this study are considered a high alarm for initiating Egyptian antimicrobial stewardship and rationalizing antibiotic use. The shift in AMR pattern for these species, ranging from increased resistance to susceptibility, should have an impact on choosing the first-line antimicrobial in practice.

### Strengths and limitations

To our knowledge, this is the first study to compare AMR before and after the COVID-19 pandemic in Egypt, covering 16 governorates. Moreover, it is the first study illustrating the MDR and XDR distribution pre- and post-COVID-19 pandemic across Egypt. Different types of specimens were collected from different geographical regions in Egypt and over different seasons. For controlling these confounders, mixed-effect linear regression models were used for the most commonly used antibiotics in Egypt as a first-line treatment, aligning with IDSA 2023 guidance^19^. However, there was a limitation in our study as, due to the paucity of some antibiotic kits, not all antibiotics were tested, such as ceftazidime-avibactam, daptomycin, and teicoplanin. The small sample size of *K. pneumoniae* culture results might hinder the discovery of more resistance patterns. Another limitation is that the study relied primarily on culture results collected from Mabaret Al-Asafra laboratories and affiliated facilities in 16 governorates, potentially introducing sampling bias towards patients seeking medical care within this network. This may not fully represent the diverse healthcare landscape across Egypt, leading to limited generalizability of the findings. Additionally, the selection of specific bacteria (*K. pneumoniae, E. coli, A. baumannii*, and MRSA) for assessing antimicrobial resistance may not encompass the full spectrum of pathogens encountered clinically. Furthermore, variations in antimicrobial susceptibility testing methodologies across laboratories could affect result consistency.

## Conclusions

The study analyzing AMR patterns pre and post the COVID-19 pandemic has led to some important conclusions. First, the COVID-19 pandemic had a noticeable impact on AMR dynamics, as shown by changes in the resistance profiles of bacterial strains such as *K. pneumoniae, A. baumannii, E. coli*, and MRSA. This rise in resistance highlights the need to implement effective antimicrobial stewardship initiatives and surveillance programs to control the spread of resistant pathogens. The study highlights the significance of acknowledging the variability in AMR patterns over time and geography. Differences in resistance profiles between regions require specific interventions and targeted surveillance to effectively manage AMR. Furthermore, it is crucial to understand the temporal variations in resistance patterns to implement customized and timely interventions to address emerging trends and prevent the spread of resistant strains. The research highlights the importance of continuing to observe and monitor AMR patterns. This helps to keep track of any changes and identify potential threats that may arise in the future. Consistent evaluation of resistance profiles is crucial to inform treatment guidelines, facilitate antimicrobial stewardship initiatives, and ensure that public health is protected from the harmful effects of AMR.

### Supplementary Information

Supplementary Tables.

## Data Availability

The datasets used and/or analyzed during the current study are available from the corresponding author on reasonable request.

## Abbreviations

*A. baumannii*: *Acinetobacter baumannii*
AMR: Antimicrobial resistance
CLSI: Clinical and Laboratory Standards Institute
COVID-19: Coronavirus disease 2019
*E. coli*: *Escherichia coli*
ESBL: Extended spectrum beta-lactamase
GIS: Geographic information system
HAIs: Hospital-acquired infections
ICU: Intensive care unit
IDSA: Infectious Diseases Society of America
IRB: Institutional Review Board
*K. pneumoniae*: *Klebsiella pneumoniae*
MDR: Multidrug-resistant
MRSA: *Methicillin-resistant* *staphylococcus aureus*
OR: Odds ratio
PDR: Pan-drug-resistant
SAM: Ampicillin-sulbactam
TZP: Piperacillin/tazobactam
XDR: Extensively drug-resistant
