# Supplementary material for: Antimicrobial resistance burden pre and post-COVID-19 pandemic with mapping the multidrug resistance in Egypt: a comparative cross-sectional study
Source: Sci Rep. 2024 Mar 26;14:7176. doi: 10.1038/s41598-024-56254-4 (PMC10966009; doi:10.1038/s41598-024-56254-4)
Supplement: Supplementary file 1 — Supplementary Tables. [file 41598_2024_56254_MOESM1_ESM.docx]

**Antimicrobial resistance (AMR) burden pre and post COVID-19 pandemic with mapping the Multidrug resistance in Egypt: comparative cross-sectional study**

Shaimaa Abdelaziz Abdelmoneim^1,2^, Ramy Mohamed Ghazy^3^, Eman Anwar Sultan^4^, Mahmoud A. Hassaan^5^, Mohamed Anwar Mahgoub^6^

^1^ Medical research Institute, Alexandria University, Egypt

^2^Clinical research administration, Alexandria Directorate of Health Affairs, Egyptian Ministry of Health and Population, Egypt

[mri.shaimaa.m.informatics18@alexu.edu.eg](mailto:mri.shaimaa.m.informatics18@alexu.edu.eg)

0000-0002-9197-6396

^3^ Tropical Health Department, High Institute of Public Health, Alexandria University, Alexandria, Egypt

[Ramy_ghazy@alexu.edu.eg](mailto:Ramy_ghazy@alexu.edu.eg)

0000-0001-7611-706X

^4^Community Medicine Department, Faculty of Medicine, Alexandria University, Egypt

[eman.sultan@alexmed.edu.eg](mailto:eman.sultan@alexmed.edu.eg)

0000-0002-9801-0607

^5^Institute of Graduate Studies & Research, Alexandria University, Egypt

[mhassaan@alexu.edu.eg](mailto:mhassaan@alexu.edu.eg)

^6^Department of Microbiology, High Institute of Public Health, Alexandria University, Egypt

[drmohamedanwar1@alexu.edu.eg](mailto:drmohamedanwar1@alexu.edu.eg) 0000-0002-1950-2428

**Supplementary Table S1 the characteristics of the isolates**

|  | *K.pneumoniae* | | | *A.baumannii* | | | *E.coli* | | | MRSA | | |
| --- | --- | --- | --- | --- | --- | --- | --- | --- | --- | --- | --- | --- |
|  | **2019**  **N = 200** | **2022**  **N = 200** | **p-value** | **2019**  **N=325** | **2022**  **N=325** | **p-value** | **2019,**  **N=380** | **2202**  **N=380** | **p-value** | **2019**  **N=310** | **2022**  **N=310** | **p-value** |
| Specimen |  |  | **<0.001*** |  |  | **<0.001*** |  |  | **<0.001*** |  |  | **0.002*** |
| Blood | 25 (12) | 141 (71) | <0.001* | 82(25) | 212(65) | <0.001* | 27 (7) | 68 (18) | <0.001* | 13 (4) | 28 (9) | 0.020* |
| Pus | 21 (10) | 10(5) | 0.048* | 43(13) | 39(12) | 0.65 | 14 (4) | 28 (7) | 0.031* | 275 (89) | 247 (80) | 0.220 |
| Respiratory | 43 (22) | 12 (6) | <0.001* | 192(59) | 68(21) | <0.001* | 13 (3) | 4 (1) | 0.021* | 19 (6) | 35 (11) | 0.023* |
| Urine | 111 (56) | 37 (18) | <0.001* | 8(3) | 6(2) | 0.591 | 326 (86) | 280 (74) | 0.064 | 3 (1) | 0 |  |
| Seasons |  |  | **0.612** |  |  | **0.203** |  |  | **<0.001*** |  |  | **<0.001*** |
| Autumn | 36 (18) | 44 (22) | 0.375 | 59 (18) | 42 (13) | 0.092 | 40 (10) | 121 (32) | <0.001* | 60 (2) | 133 (43) | <0.001* |
| spring | 46 (23) | 52 (26) | 0.687 | 83 (26) | 91 (28) | 0.540 | 142 (37) | 88 (23) | <0.001* | 119 (38) | 54 (17) | <0.001* |
| summer | 50 (25) | 44 (22) | 0.532 | 89 (27) | 102 (31) | 0.341 | 97 (26) | 59 (16) | 0.002* | 77 (25) | 47 (15) | 0.102 |
| Winter | 67 (34) | 61 (30) | 0.590 | 94 (29) | 90 (28) | 0.760 | 101 (27) | 112 (29) | 0.451 | 54 (17) | 76 (25) | 0.455 |
| Geographical distribution |  |  | **0.751** |  |  | **0.024*** |  |  | **0.012*** |  |  | **<0.001*** |
| Metropolitan | 65 (33) | 60 (30) | 0.530 | 171(52) | 169 (52) | 0.912 | 147 (39) | 122 (32) | 0.123 | 184 (59) | 137 (44) | 0.01* |
| Lower Egypt | 131(66) | 138(69) | 0.678 | 152(47) | 144 (44) | 0.642 | 228 (60) | 241 (63) | 0.540 | 113 (36) | 171 (55) | 0.001* |
| Upper Egypt | 2 (1) | 3 (1) | 0.655 | 2 (1) | 12 (4) | 0.013* | 5 (1) | 17 (5) | 0.014* | 13 (4) | 2 (1) | 0.005* |

**Supplementary Table S2 K.pneumoniae resistance distribution pre and post COVID-19**

| Antibiotic | 2019 pre COVID-19 era (N= 200) | | | 2022 post COVID-19 era (N= 200) | | | p-value |
| --- | --- | --- | --- | --- | --- | --- | --- |
|  | **Resistant** | **Intermediate** | **Sensitive** | **Resistant** | **Intermediate** | **Sensitive** |  |
|  | **n(%)** | | | **n(%)** | | |  |
| Amikacin | 25 (13) | 2 (1.0) | 172(86) | 130 (65) | 3 (1.5) | 68 (34) | <0.001* |
| AMC | 125 (63) | 1 (0.5) | 73 (37) | 181 (90) | 3 (1.5) | 17 (8.5) | <0.001* |
| Ampicillin | 199 (100) | 0 | 0 | 200(99.5) | 0 | 1 (0.5) | 0.319 |
| SAM | 128 (64) | 0 | 71 (36) | 182 (92) | 0 | 19 (9.5) | <0.001* |
| Cefepime | 107 (54) | 0 | 92 (46) | 175 (87) | 0 | 26 (13) | <0.001* |
| Cefotaxime | 107 (54) | 0 | 92 (46) | 176 (88) | 0 | 25 (12) | <0.001* |
| Cefoxitin | 64 (32) | 13 (6.5) | 120(60) | 151 (75) | 0 | 50 (25) | <0.001* |
| Ceftazidime | 106 (53) | 0 | 93 (47) | 176 (88) | 0 | 25 (12) | <0.001* |
| Ceftriaxone | 107 (54) | 0 | 92 (46) | 177 (88) | 0 | 24 (12) | <0.001* |
| Ciprofloxacin | 87 (44) | 1 (0.5) | 109(55) | 141 (70) | 2 (1.0) | 58 (29) | <0.001* |
| Doxycycline | 151 (76) | 0 | 38 (19) | 190 (95) | 0 | 11 (5.5) | <0.001* |
| Ertapenem | 33 (17) | 1 (0.5) | 162 (81) | 138(69) | 0 | 63 (31) | <0.001* |
| Fosfomycin | 52 (26) | 1 (0.5%) | 58(29) | 37 (18) | 0 | 0 | <0.001* |
| Gentamycin | 44 (22) | 2 (1.0) | 150(75) | 140 (70) | 3 (1.5) | 58 (29) | <0.001* |
| Imipenem | 32 (16) | 0 | 164(82) | 131 (65) | 0 | 70 (35) | <0.001* |
| Levofloxacin | 82 (41) | 1 (0.5%) | 114(57) | 138 (69) | 4 (2.0) | 59 (29) | <0.001* |
| Meropenem | 32 (16) | 0 | 165(83) | 133 (66) | 0 | 68 (34) | <0.001* |
| Moxifloxacin | 82 (41) | 1 (0.5%) | 114(57) | 140 (70) | 5 (2.5) | 56 (28) | <0.001* |
| Nitrofurantoin | 69 (35) | 6 (3.0) | 37 (19) | 42 (21) | 0 | 0 | <0.001* |
| Ofloxacin | 83 (42) | 2 (1.0) | 112(56) | 143 (71) | 5 (2.5) | 53 (26) | <0.001* |
| TZP | 69 (35) | 2 (1.0) | 126(63) | 143 (71) | 3 (1.5) | 55 (27) | <0.001* |
| SXT | 112 (56) | 0 | 85 (43) | 180 (90) | 0 | 21 (10) | <0.001* |

**Supplementary Table S3 K.pneumoniae Logistic regression (geographical distribution is the random intercept)**

|  | NIT | Doxycycline | Ceftazidime | Cefepime | Amikacin | Levofloxacin | Ciprofloxacin | Moxifloxacin | Ertapenem | IPM | Meropenem |
| --- | --- | --- | --- | --- | --- | --- | --- | --- | --- | --- | --- |
| Adjusted OR (95%CI) | | | | | | | | | | | |
| Year  (Ref: 2019) | 1.29  (1.15-1.44)  p<0.001* | 1.15  (1.07 -1.21)  p<0.001* | 1.16 (1.08 -1.24)  p<0.001* | 1.16  (1.08 -1.24) p<0.001 * | 1.27 (1.19 -1.34)  P<0.001* | 1.18 (1.09 -1.28) p<0.001* | 1.16 (1.07 -1.25) p<0.001* | 1.19 (1.10-1.28) p<0.001* | 1.23 (1.15 - 1.31)  p<0.001* | 1.18 (1.11-1.26)  p<0.001* | 1.20 (1.12-1.27)  p<0.001* |
| Specimen (Ref: pus) | | | | | | | | | | | |
| Blood | .. | 0.91  (0.71-1.18)  p=0.471 | 1.06 (0.75 -1.51) p=0.720 | 1.05  (0.74 -1.48)  p=0.711 | 1.56  (1.15 -2.14)  p=0.010* | 1.02 (0.69 - 1.52)  p=0.900 | 1.09 (0.74 -1.61)  p=0.667 | 1.05 (0.74 - 1.55) p=0.812 | 1.96 (1.43 - 2.69) p<0.001* | 2.15 (1.57 - 2.94) p<0.001* | 1.84 (1.35 -2.53) p<0.001* |
| Respiratory isolates | .. | 0.78  (0.60-1.03)  p=0.081 | 0.81 (0.56 - 1.17) p=0.262 | 0.81  (0.56-1.17)  p=0.261 | 1.12  (0.80 -1.56)  p=0.501 | 1.06 (0.69 - 1.52)  p=0.793 | 1.05 (0.69 -1.62) p=0.824 | 1.06 (0.69 - 1.60) p=0.784 | 1.10 (0.78 - 1.55) p=0.575 | 1.04 (0.74 - 1.46) p=0.804 | 0.91 (0.65 -1.28) p=0.610 |
| Urine | .. | 1.42  (1.12-1.81)  p=0.011* | 0.64 (0.46 - 0.89)  p=0.010* | 0.65 (0.46 -0.90)  p=0.011* | 0.70  (0.52 -0.94)  p=0.020* | 0.85  (0.58 -1.24)  p=0.411 | 0.89 (0.61 -1.29) p=0.545 | 0.85 (0.58 - 1.24) p=0.401 | 0.82 (0.61 - 1.12) p=0.221 | 0.85 (0.63 - 1.16) p=0.310 | 0.75 (0.55 -1.01) p=0.080 |
| Seasons (Ref: Autum) | | | | | | | | | | | |
| Summer | 0.73  (0.41-1.30)  p=0.28 | 0.94  (0.77-1.13) p=0.524 | 1.04 (0.81 -1.24) p=0.742 | 1.01 (0.78 -1.19) p=0.932 | 0.87  (0.69 -1.10)  p=0.255 | 1.00 (0.78 -1.40) p=0.987 | 1.02 (0.76 -1.36) p=0.911 | 0.99 (0.74 - 1.33) p=0.962 | 0.94 (0.74 - 1.19) p=0.611 | 0.85 (0.68 - 1.08) p=0.191 | 0.87 (0.69 -1.10) p=0.252 |
| Spring | 0.85  (0.49-1.52)  p=0.57 | 1.03  (0.85-1.25) p=0.785 | 1.23 (0.95 -1.59) p=0.110 | 1.22 (0.94 -1.57) p=0.132 | 0.80  (0.64 -1.02)  p=0.061 | 1.04 (0.78 - 1.40) p=0.778 | 1.14 (0.85 -1.52) p=0.392 | 1.05 (0.78 - 1.4) p=0.754 | 0.83 (0.65 - 1.05) p=0.122 | 0.81 (0.64 - 1.02) p=0.081 | 0.82 (0.65 -1.04) p=0.100 |
| Winter | 0.86  (0.51-1.48)  p=0.57 | 1.09  (0.92-1.31) p=0.326 | 1.29 (1.02 -1.64) p=0.033* | 1.31 (1.03 -1.66) p=0.031* | 1.18  (0.95 -1.46)  p=0.140 | 1.46 (1.11 -1.92) p=0.011* | 1.45 (1.17 -2.01) p=0.002* | 1.46 (1.11 - 1.92) p=0.010* | 1.23 (0.98 - 1.53) p=0.070 | 1.19 (0.95 - 1.47) p=0.110 | 1.2 (0.96 -1.49) p=0.102 |

**Supplementary Table S4 A.baumannii resistance distribution pre and post COVID-19**

| Antibiotic | 2019 pre COVID-19 era (N=325) | | | 2022 post COVID-19 era (N=325) | | | p-value |
| --- | --- | --- | --- | --- | --- | --- | --- |
|  | Resistant | Intermediate | Sensitive | Resistant | Intermediate | Sensitive |  |
|  | n(%) | | | n(%) | | |  |
| Amikacin | 233 (71.7) | 2 (0.6) | 90 (27.7) | 306 (94.2) | 2 (0.6) | 17 (5.2) | <0.001* |
| SAM | 236 (72.6) | 0 | 89 (27.4) | 304 (93.5) | 0 | 21 (6.5) | <0.001* |
| Cefepime | 266 (81.8) | 0 | 59 (18.2) | 319 (98.0) | 0 | 6 (1.8) | <0.001* |
| Cefotaxime | 307 (94.5) | 5 (1.5) | 13 (4.0) | 322(99.1) | 0 | 3 (0.9) | 0.003* |
| Ceftazidime | 270 (83.1) | 2 (0.6) | 53 (16.3) | 322 (99.1) | 0 | 3 (0.9) | <0.001* |
| Ceftriaxone | 305 (93.8) | 4 (1.2) | 16 (4.9) | 322 (99.1) | 0 | 3 (0.9) | <0.001* |
| Ciprofloxacin | 238 (73.2) | 9 (2.8) | 78 (24.0) | 289 (88.9) | 4 (1.2) | 32 (9.8) | <0.001* |
| Colistin | 0 | 0 | 325 (100) | 1 (0.3) | 0 | 323(99) | 0.316 |
| Doxycycline | 227 (69.8) | 0 | 98 (30.2) | 301 (92.6) | 0 | 24 (7.4) | <0.001* |
| Gentamycin | 201 (61.8) | 2 (0.6) | 122 (37.5) | 272 (83.7) | 6 (1.80) | 47 (14.5) | <0.001* |
| Imipenem | 203(62.5) | 2 (0.6) | 120 (36.9) | 273 (84.0) | 5 (1.5) | 47 (14.5) | <0.001* |
| Levofloxacin | 236 (72.6) | 5 (1.5) | 84 (25.8) | 285 (87.7) | 3 (0.9) | 37 (11.4) | <0.001* |
| Meropenem | 225 (69.2) | 10 (3.1) | 90 (27.7) | 290 (89.2) | 5 (1.5) | 30 (9.2) | <0.001* |
| TZP | 217 (66.8) | 4 (1.2) | 104 (32.0) | 309 (95.0) | 4 (1.2) | 12 (3.7) | <0.001* |
| Tigecycline | 1 (0.3) | 0 | 316 (99.7) | 7 (2.2) | 1 (0.3) | 305 (97) | 0.059 |
| SXT | 241 (74.2) | 1 (0.3) | 83 (25.5) | 310 (95.4) | 0 | 15 (4.6) | <0.001* |

* Significance between the resistance pre and post the COVID-19 era, SAM: ampicillin-sulbactam, TZP: Piperacillin-tazobactam, SXT: Trimethoprim / Sulfamethoxazole

**Supplementary Table S5 A.baumannii logistic regression (mixed effect model, geographical distribution used as random intercept)**

|  | Doxycycline | Ceftazidime | Cefepime | Levofloxacin | Ciprofloxacin | TZP | Amikacin | IPM | Meropenem |
| --- | --- | --- | --- | --- | --- | --- | --- | --- | --- |
| Adjusted OR 95%CI | | | | | | | | | |
| Year  Ref: 2019 | 1.73 (1.72 - 1.73)  p<0.001* | 2.75 (1.86 - 4.07)  p<0.001* | 2.26 (1.70 -3.01)  p<0.001* | 1.36 (1.16 -1.59)  p<0.001* | 1.42 (1.20 -1.68)  p<0.001* | 2.13 (2.12 -2.13)  p<0.001* | 1.82 (1.81-1.84)  p<0.001* | 1.48 (1.48 -1.49)  p<0.001* | 1.51 (1.50 - 1.51) p<0.001* |
| Specimen (Ref: pus) | | | | | | | | | |
| Blood | 0.92 (0.46 - 1.86)  p=0.821 | .. | .. | 1.19 (0.60 - 2.37)  p=0.611 | 1.17 (0.58 - 2.39)  p=0.660 | 2.53 (1.21 - 5.26)  p=0.011* | 3.12 (1.52 -6.41)  p=0.002* | 1.59 (0.89 -2.84) p= 0.112 | 2.31 (1.19 - 4.49) p=0.010* |
| Respiratory isolates | 0.79 (0.41 - 1.54)  p=0.481 | .. | .. | 0.90 (0.47 - 1.73)  p=0.751 | 1.01 (0.51 - 1.98)  p=0.987 | 1.16 (0.62 - 2.18)  p=0.63 | 1.70 (0.91 -3.17)  p=0.091 | 1.39 (0.79 -2.42) p=0.241 | 1.43 (0.78 - 2.61) p=0.242 |
| Urine | 0.98 (0.22 - 4.38)  p=0.987 | .. | .. | 0.20 (0.05 - 0.74)  p=0.020* | 0.19 (0.05 - 0.73)  p=0.010* | 0.53 (0.10 - 2.81)  p=0.45 | 0.30 (0.08 -1.14)  p=0.072 | 0.21 (0.06 -0.80)  p=0.020* | 0.33 (0.07 - 1.46) p=0.143 |
| Seasons (Ref: Autum) | | | | | | | | | |
| Summer | 0.96 (0.49 - 1.89)  p=0.910 | 1.54 (0.61 - 3.87)  p=0.360 | 1.87 (0.80 -4.37)  p=0.150 | 1.24 (0.62 - 2.47)  p=0.530 | 1.36 (0.66 - 2.80)  p=0.402 | 1.47 (0.75 - 2.90)  p=0.262 | 0.72 (0.34 -1.53)  p=0.390 | 1.47 (0.82 -2.63)  p=0.190 | 2.64 (1.33 - 5.22) p= 0.010* |
| Spring | 0.88 (0.45 - 1.72)  p=0.712 | 1.07 (0.44 – 2.60)  p=0.874 | 1.33 (0.59 -3.00) p= 0.491 | 1.03 (0.52 - 2.02)  p=0.943 | 1.09 (0.54 - 2.19)  p=0.818 | 1.20 (0.61 - 2.35)  p=0.592 | 0.69 (0.33 -1.47)  p=0.341 | 1.36 (0.75 -2.43)  p=0.300 | 1.74 (0.91 - 3.31) p=0.091 |
| Winter | 0.69 (0.37 - 1.32)  p=0.260 | 0.76 (0.33 - 1.73)  p=0.503 | 0.81 (0.38 -1.72) p=0.592 | 0.58 (0.31 - 1.08)  p=0.080 | 0.55 (0.29 - 1.05)  p=0.072 | 1.10 (0.58 - 2.09)  p=0.774 | 0.45 (0.22 -0.92)  p=0.03* | 0.96 (0.55 -1.68) p=0.887 | 0.82 (0.46 - 1.48) p=0.511 |

**Supplementary Table S6 E.coli resistance distribution pre and post COVID-19**

| Antibiotic | 2019 pre COVID-19 era  (N=380) | | | 2022 post COVID-19 era  (N=380) | | | p-value* |
| --- | --- | --- | --- | --- | --- | --- | --- |
|  | Resistant | Intermediate | Sensitive | Resistant | Intermediate | Sensitive |  |
|  | n (%) | | | n (%) | | |  |
| Amikacin | 8 (2) | 6 (2) | 364 (96) | 31 (8) | 1 (0.3) | 347 (91.3) | 0.001* |
|  | 189 (50) | 0 | 191 (50) | 250 (66) | 0 | 130 (34) | <0.001* |
| SAM | 189 (50) | 2 (0.5) | 189 (49.5) | 251 (66) | 0 | 129 (34) | <0.001* |
| Cefoxitin | 104 (27) | 10 (3) | 266 (70) | 107 (28) | 10 (3) | 263 (69) | 0.971 |
| Cefotaxime | 173 (45.5) | 2 (0.5) | 205 (54) | 222 (58) | 0 | 158 (42) | 0.001* |
| Ceftriaxone | 173 (45.5) | 3 (0.8) | 204 (53.2) | 223 (59) | 0 | 157 (41) | <0.001* |
| Ceftazidime | 172 (45.3) | 2 (0.5) | 206 (54.2) | 223 (59) | 0 | 157 (41) | <0.001* |
| Cefepime | 172 (45) | 0 | 208 (55) | 219 (58) | 0 | 161 (42) | <0.001* |
| Ciprofloxacin | 169 (45) | 8 (2) | 203 (53) | 142 (37.4) | 1 (0.3) | 237 (62.4) | 0.004* |
| Doxycycline | 315 (83) | 0 | 65 (17) | 372 (98) | 0 | 8 (2) | <0.001* |
| Ertapenem | 3 (0.8) | 1 (0.3) | 376 (98.9) | 28 (7) | 2 (1) | 350 (92) | <0.001* |
| Fosfomycin | 31 (8.2) | 1 (0.3) | 288 (76) | 190 (50.0) | 1 (0.3) | 89 (23.4) | <0.001* |
| Gentamycin | 63 (16.6) | 6 (1.6) | 311 (81.8) | 91 (24) | 3 (1) | 286 (75) | 0.028* |
| Imipenem | 4 (1) | 0 | 376 (99) | 23 (6) | 0 | 357 (94) | <0.001* |
| Levofloxacin | 168 (44) | 0 | 212 (56) | 141 (37) | 0 | 238 (63) | 0.049* |
| Moxifloxacin | 167 (44) | 0 | 213 (56) | 143 (38) | 0 | 237 (62) | 0.076 |
| Meropenem | 3 (0.8) | 0 | 377 (99.2) | 24 (6.3) | 1 (0.3) | 354 (93.4) | <0.001* |
| Nitrofurantoin | 70 (18) | 26 (7) | 230 (61) | 175 (46) | 11 (3) | 94 (25) | <0.001* |
| Norfloxacin | 163 (43) | 4 (1) | 159 (42) | 87 (23) | 0 | 193 (51) | <0.001* |
| Ofloxacin | 168 (44) | 13 (3) | 198 (52) | 146 (38.4) | 1 (0.3) | 233 (61.3) | 0.001* |
| TZP | 45 (12) | 16 (4) | 319 (84) | 90 (24) | 5 (1) | 285 (75) | <0.001* |
| Tigecycline | 0 | 0 | 52 (100) | 0 | 0 | 92 (100) | - |
| SXT | 212 (55.8) | 2 (0.5) | 166 (43.7) | 254 (67) | 0 | 126 (33) | 0.002* |

AMC Amoxicillin clavulanate, SAM Amp-sulbactam, TZP piperacillin-tazobactam, SXT Trimethoprim / Sulfamethoxazole

**Supplementary Table S7 E.coli logistic regression (geographical distribution is the random intercept)**

|  | Fosfomycin | NIT | SAM | Cefoxitin | Ceftriaxone | Ceftazidime | Cefepime | Levofloxacin | Ciprofloxacin | TZP | IPM | Meropenem |
| --- | --- | --- | --- | --- | --- | --- | --- | --- | --- | --- | --- | --- |
| Adjusted OR (95%CI) | | | | | | | | | | | | |
| Year | 1.44  (1.37-1.50)  P<0.001* | 1.28  (1.21 – 1.34)  p<0.001* | 1.11 (1.06 -1.1) p<0.001* | 0.97 (0.93-1.02)  p=0.220 | 1.08 (1.03 -1.14)  p=0.002* | 1.09 (1.03 -1.14) p=0.001* | 1.08 (1.03-1.14) p=0.002* | 0.94 (0.89-0.98)  p=0.02* | 0.93 (0.89-0.98) p=0.01* | 1.02 (0.97-1.07) p=0.430 | 1.03 (1.01-1.05)  p=0.002* | 1.03 (1.01-1.05)  p<0.001* |
| Specimen (RF: respiratory isolates) | | | | | | | | | | | | |
| Blood | .. | .. | 0.52 (0.31-0.85)  p=0.010* | 0.81 (0.51 - 1.26)  p=0.362 | 1.08 (0.64 -1.82)  p=0.766 | 1.08 (0.64 -1.81)  p=0.774 | 0.99 (0.59 -1.67) p= 0.970 | 0.99 (0.60 -1.68)  p=0.990 | 0.97 (0.58 -1.64)  p=0.911 | 1.70 (1.00 -2.87)  p=0.050* | 1.33 (1.11-1.60)  p=0.002* | 1.31 (1.09-1.58)  p=0.004* |
| Pus | .. | .. | 0.59 (0.34 - 1.02)  p=0.061 | 0.65 (0.40 -1.06) p=0.087 | 1.10 (0.63 -1.92) p=0.734 | 1.10 (0.63 -1.92)  p=0.731 | 1.16 (0.66 -2.02)  p=0.611 | 1.30 (0.74 -2.27)  p=0.350 | 1.33 (0.77 -2.33)  p=0.300 | 1.28 (0.72 -2.24)  p=0.390 | 1.03 (0.84 -1.25) p=0.800 | 1.02 (0.83 -1.24)  p=0.861 |
| Urine | .. | .. | 0.40 (0.25 - 0.64)  p<0.001* | 0.37  (0.24 - 0.55)  p<0.001* | 0.86 (0.53 -1.39)  p=0.551 | 0.86 (0.53 -1.39)  p=0.542 | 0.85 (0.53 -1.38) p=0.531 | 0.89 (0.56 -1.45)  p=0.651 | 0.91 (0.57 -1.48)  p=0.711 | 0.72 (0.44 -1.17)  p=0.192 | 1.01 (0.84 -1.19)  p=0.940 | 1.01 (0.84 -1.20)  p=0.932 |
| Seasons (RF: Autum) | | | | | | | | | | | | |
| Summer | 0.95  (0.78-1.15)  p=0.60 | 0.97  (0.77 - 1.21)  p=0.791 | 0.95  (0.76 - 1.19) p=0.697 | 1.01 (0.83 - 1.22)  p=0.911 | 0.94 (0.75 -1.18)  p=0.597 | 0.93 (0.74 -1.14)  p=0.574 | 0.94 (0.75 -1.18)  p=0.600 | 0.92 (0.73 -1.16)  p=0.470 | 0.95 (0.76 -1.20) p=0.694 | 0.94 (0.75 -1.19)  p=0.625 | 1.03 (0.95 -1.12)  p=0.445 | 1.03 (0.94 -1.11)  p=0.522 |
| Spring | 0.75  (0.62-0.91)  p=0.004* | 0.84  (0.68 - 1.05)  P=0.141 | 1.23 (1.00 - 1.52) p= 0.040* | 0.89 (0.74 - 1.22)  p=0.223 | 1.20 (.96 -1.48) p=0.100 | 1.20 (0.96 -1.49) p=0.091 | 1.17 (0.94 -1.46) p=0.145 | 0.87 (0.70 -1.08)  p=0.216 | 0.89 (0.72 -1.10) p=0.281 | 1.14 (0.91 -1.41)  p=0.241 | 1.11 (1.03 -1.20)  p=0.010* | 1.10 (1.02 -1.19)  p=0.020* |
| Winter | 0.58  (0.48-0.69)  p<0.001* | 0.69  (0.56 - 0.84)  p<0.001* | 1.05 (0.86 - 1.28)  p=0.621 | 0.90 (0.76 - 1.08) p=0.28 | 1.04 (0.84 -1.27)  p=0.730 | 1.03 (0.83 -1.26)  p=0.785 | 1.05 (0.85 -1.29)  p=0.634 | 0.92 (0.75-1.13), p=0.454 | 0.93 (0.76 -1.15)  p=0.532 | 1.28 (1.04 - 1.58) p=0.020* | 1.02 (0.94 -1.09)  p=0.650 | 1.01 (0.84 -1.09) p=0.831 |

NIT Nitrofurantoin, SAM Ampicillin-sulbactam , TZP Piperacillin-tazobactam, IPM Imipenem

**Supplementary Table S8 MRSA resistance distribution pre and post COVID-19**

| Antibiotic | 2019 pre COVID-19 era  (N=310) | | | 2022 post COVID-19 era  (N=310) | | | p-value* |
| --- | --- | --- | --- | --- | --- | --- | --- |
|  | Resistant | Intermediate | Sensitive | Resistant | Intermediate | Sensitive |  |
|  | n(%) | | | n (%) | | |  |
| Azithromycin | 47 (15.20) | 2 (0.60) | 258 (83.20) | 134 (43.20) | 0 | 176 (56.80) | <0.001* |
| Ciprofloxacin | 42 (13.50) | 16 (5.20) | 252 (81.30) | 61 (19.70) | 10 (3.20) | 239 (77.10) | 0.073 |
| Clindamycin | 47 (15.20) | 0 | 260 (83.80) | 120 (38.70) | 0 | 190 (61.30) | <0.001* |
| Doxycycline | 97 (31.30) | 22 (7.10) | 191 (61.60) | 215 (69.40) | 38 (12.3) | 57 (18.40) | <0.001* |
| Gentamycin | 156 (50.30) | 4 (1.30) | 149 (48.06) | 146 (47.10) | 3 (1.00) | 161 (51.90) | 0.585 |
| Levofloxacin | 37 (11.90) | 5 (1.60) | 268 (86.50) | 62 (20.00) | 0 | 248 (80.00) | 0.002* |
| Linezolid | 1 (0.30) | 0 | 306 (98.70) | 0 | 0 | 309 (99.67) | 0.365 |
| Moxifloxacin | 34 (11.00) | 7 (2.30) | 269 (86.80) | 62 (20.00) | 0 | 248 (80.00) | <0.001* |
| Ofloxacin | 41 (13.20) | 9 (2.90) | 260 (83.90) | 61 (19.70) | 9 (2.90) | 240 (77.40) | 0.094 |
| Tigecycline | 0 | 0 | 307 (99.00) | 0 | 0 | 310 (100) | 0.083 |
| SXT** | 172 (55.50) | 1 (0.30) | 137 (44.20) | 157 (50.60) | 0 | 153 (49.40) | 0.277 |
| Vancomycin | 0 | 0 | 307 (99.00) | 0 | 0 | 310 (100) | 0.083 |

**SXT Trimethoprim / Sulfamethoxazole

**Supplementary Table S9 MRSA Logistic regression (geographical distribution is the random intercept)**

|  | SXT | Clindamycin | Doxycycline | Levofloxacin | Ciprofloxacin | Moxifloxacin | Linezolid |
| --- | --- | --- | --- | --- | --- | --- | --- |
| Adjusted OR (95%CI) | | | | | | | |
| Year  Ref: (2019) | 0.98 (0.96 - 1.02)  p=0.420 | 1.07 (1.05 - 1.10)  p<0.001* | 1.15 (1.12 - 1.1 8)  p<0.001* | 1.02 (1.00 - 1.04)  p=0.020* | 1.02 (0.99 - 1.04) p=0.091 | 1.03 (1.01 - 1.05)  p=0.010* | 0.99 (0.99 - 1.00)  p= 0.522 |
| Specimen (Ref: respiratory isolates) | | | | | | | |
| Blood | 0.94 (0.77 - 1.16)  p=0.581 | 1.19 (1.00 - 1.41)  p =0.040* | 0.97 (0.81 - 1.18)  p=0.812 | 1.16 (1.00 - 1.34)  p=0.050 | 1.19 (1.02 -1.38)  p=0.030* | 1.16 (1.00 - 1.16) p=0.040* | 1.00 (0.98 -1.02)  p=0.981 |
| Pus | 1.00 (0.87 - 1.16) p=0.941 | 0.93 (0.82 - 1.05)  p=0.242 | 0.93 (0.82 - 1.07)  p=0.322 | 0.93 (0.84 -1.04)  p=0.191 | 0.93 (0.84 - 1.04)  p=0.232 | 0.93 (0.84 -1.03)  p=0.174 | 1.00 (0.99 -1.00)  p=0.910 |
| Urine | 1.57 (0.87 - 2.80) p=0.133 | .. | 1.85 (1.10 - 3.04)  p=0.020* | 0.82 (0.49 - 1.38)  p=0.452 | 1.12 (0.72 -1.73)  p=0.604 | 0.83 (0.50 - 1.38)  p=0.470 | .. |
| Seasons (Ref: Autum) | | | | | | | |
| Summer | 1.02 (0.91 -1.12) p=0.697 | 0.97 (0.86 - 1.06)  p=0.380 | 1.02 (0.92 - 1.14) p= 0.661 | 1.06 (0.97 - 1.16)  p=0.166 | 1.06 (0.97 -1.16)  p=0.190 | 1.06 (0.97 -1.19)  p=0.194 | 0.99 (0.99 -1.01)  p=0.880 |
| Spring | 1.04 (0.93 - 1.16)  p=0.443 | 0.97 (0.88 - 1.07)  p=0.523 | 0.87 (0.79 - 0.96)  p=0.010* | 0.98 (0.91- 1.07)  p=0.797 | 0.99 (0.92 -1.08) p=0.987 | 0.98 (0.91 -1.06)  p=0.675 | 1.00 (0.99 -1.00) p=0.266 |
| Winter | 1.04 (0.92 - 1.16) p=0.511 | 1.00 (0.91 -1.11) p=0.923 | 1.04 (0.93 - 1.15)  p=0.430 | 0.99 (0.91 - 1.07) p=0.796 | 0.98 (0.90 -1.08)  p=0.787 | 0.99 (0.91 - 1.07)  p=0.840 | 1.00 (0.99 - 1.01) p=0.954 |
